# Dezocine modulates the reinstatement of conditioned place preference in morphine-dependent rats via the dopamine reward circuitry

**DOI:** 10.3389/fnins.2025.1507747

**Published:** 2025-02-18

**Authors:** Yan He, Zhi-Sheng Piao, Yi Jia, Hui-Qi Wu, Xiao-Qiang Wang, Wei-Feng Yu, Fei-Xiang Wu

**Affiliations:** ^1^Department of Anesthesiology, Eastern Hepatobiliary Surgery Hospital Affiliated to Naval Medical University, Shanghai, China; ^2^Department of Anesthesiology, Fuzhou First Hospital Affiliated to Fujian Medical University, Fuzhou, China; ^3^Department of Critical Care Medicine, Eastern Hepatobiliary Surgery Hospital Affiliated to Naval Medical University, Shanghai, China; ^4^Department of Anesthesiology, Ren Ji Hospital of Shanghai Jiao Tong University, Shanghai, China; ^5^Key Laboratory of Anesthesiology (Shanghai Jiao Tong University), Ministry of Education, Shanghai, China; ^6^Department of Anesthesiology, The First Affiliated Hospital of Wenzhou Medical University, Wenzhou, China

**Keywords:** opioid-related disorders, morphine dependence, dezocine, therapeutics, substance withdrawal syndrome

## Abstract

**Introduction:**

Opioid addiction is a significant public health issue, with existing treatments such as buprenorphine and methadone exhibiting limitations, including side effects and insufficient prevention of relapse. Novel therapeutic strategies are needed to address these challenges. This study investigates the potential of dezocine in reducing addiction-related behaviors and preventing relapse.

**Methods:**

A morphine-induced conditioned place preference (CPP) model was established in rats to evaluate the effect of dezocine on addiction-related behaviors. Behavioral assessments were conducted to measure withdrawal symptoms and CPP reinstatement. To explore the underlying mechanism, Western blot (WB) and immunofluorescence (IF) were used to quantify the expression of phosphorylated DARPP32 (p-DARPP32) and DOPA decarboxylase (DDC) in reward-related brain regions, including the nucleus accumbens (NAc), ventral tegmental area (VTA), hippocampus (HP), and prefrontal cortex (PFC).

**Results:**

Dezocine significantly reduced withdrawal symptoms and prevented CPP reinstatement, indicating its potential to alleviate addiction behaviors. Western blotting and immunofluorescence analysis revealed that dezocine increased p-DARPP32 expression in the NAc, VTA, HP, and PFC, without altering DDC levels.

**Discussion:**

These findings suggest that dezocine may exert its therapeutic effects by inhibiting kappa opioid receptor activation and enhancing dopamine signaling in reward-related brain circuitry. The increase in p-DARPP32 expression in key brain regions supports this mechanism, providing insights into the potential clinical application of dezocine for managing opioid addiction. Dezocine represents a promising candidate for opioid addiction treatment, with the ability to control withdrawal symptoms and prevent relapse.

## 1 Introduction

Opioid addiction poses a significant challenge to public health, exacerbated by the increased focus on pain management and the widespread illicit use of opioids. Substance use disorder has become a global health concern, with ~35 million individuals requiring treatment services (Jessica and Jeffrey, 2022). In particular, opioid misuse poses a critical challenge in the United States. In 2016, more than 42,000 Americans died from opioid overdose, and this number continues to rise (Volkow et al., 2019a). This problem significantly affects the health of those with addiction, increases social burdens, and introduces various factors of instability.

Drug addiction and mental dependence are classified as chronic, protracted encephalopathies (Evaristo, 2022). Addiction progresses through three stages: abuse and addiction, withdrawal with negative emotions, and cravings leading to relapse. Each stage involves varying changes in neural adaptability and plasticity, resulting in diverse physical and mental dependence symptoms. The primary reason for recurring substance abuse is linked to mental dependence, influenced by various social, environmental, and psychological factors (Sheng et al., 2005; George and Michel, 2008). Inhibition of γ-aminobutyric acid (GABA)ergic interneurons in the ventral tegmental area (VTA) during opioid use, leading to the disinhibition of dopaminergic neurons in the VTA and activation of the reward circuitry in the nucleus accumbens (NAc), has been reported as one of the mechanisms involved in opioid addiction (John et al., 2020). During opioid withdrawal, the dynorphin/kappa opioid receptor (KOR) system plays a role in the hyperactivation of the brain's stress system and the resulting dopaminergic deficit (Carlezon et al., 2000). Reports suggest that down-regulating the KOR receptor system in KOR gene knockout animals alleviates withdrawal symptoms, such as reduced pleasure and disgust mediated by KOR activation (Yan et al., 2023). Additionally, KOR antagonists can block the potentiation of drug reward pathways and inhibit the reinstatement of drug-seeking behavior induced by stress (Timothy et al., 2015).

Current treatments for opioid addiction include alternatives like methadone and buprenorphine, the latter acts as a partial μ-opioid receptor (UOR) agonist and a partial KOR antagonist (Richard et al., 2018). However, both are limited by side effects and the risk of further addiction (Albert et al., 2022; Cristian et al., 2024). Thus, finding a drug that effectively addresses mental dependence and relapse prevention, while offering high safety, low addiction potential, and accessibility, is crucial for opioid addiction treatment. Dezocine has demonstrated unique pharmacological effects, including weak partial agonism at the UOR and antagonism at the KOR. This profile, which shares pharmacological similarities with buprenorphine indicates promising potential for treating opioid addiction and mental dependence. Dezocine, an analgesic used for perioperative pain, has shown a strong safety profile in clinical practice, with no instances of respiratory depression and a very low potential for addiction (Yong-Kang et al., 2023; Gordon et al., 2024). Given its unique pharmacological profile, low addiction risk and safety, dezocine could play a significant role in the clinical management of opioid addiction, potentially improving patient compliance and treatment outcomes. This study aims to evaluate the effects of dezocine on mitigating withdrawal syndrome in morphine-dependent rats and its preventive effect on the reinstatement of conditioned place preference (CPP) behavior. Additionally, the study preliminarily explores the central mechanisms underlying dezocine's actions, providing new insights and potentially improved therapeutic strategies for clinical use.

## 2 Materials and methods

### 2.1 Animals

Male SD adult rats (200–250 g) were obtained from the Shanghai Experimental Animal Center, China Academy of Medical Sciences. They were housed under SPF (Specific Pathogen Free) conditions at 20–24°C with 40%–60% humidity. The lighting cycle was 12 h light/dark, with food and water available ad libitum.

### 2.2 Drugs

Morphine hydrochloride injection was sourced from Shenyang First Pharmaceutical Co., Ltd., Northeast Pharmaceutical Group. Dezocine hydrochloride injection was obtained from Jiangsu Yangtze River Pharmaceutical Group Co., Ltd. Buprenorphine hydrochloride injection was acquired from Yujin Pharmaceutical Research Institute Pharmaceutical Co., Ltd. Naloxone hydrochloride injection was purchased from GuoYaoYixin Pharmaceutical Co., Ltd. Dimethyl sulfoxide (DMSO) was procured from Beijing Hanronda Technology Development Co., Ltd. (–)-trans-(1S,2S)-U50488 was sourced from Sigma-Aldrich (USA). For animal experiments, dezocine, buprenorphine, and U50488 were diluted in DMSO and normal saline. The final concentration of DMSO was maintained at 1% (v/v) to minimize potential toxicity to the rats. In the experiment, the single injection volume was controlled at 5 ml/kg. The Anti-GAPDH antibody was purchased from Cell Signaling Technology, USA. Phospho-DARPP32 (Thr34) Polyclonal Antibody was obtained from Thermo Fisher Scientific, USA. The Anti-DOPA Decarboxylase antibody was purchased from Abcam, UK. Goat Anti-Rabbit IgG H&L (HRP) was sourced from Abcam, UK. Donkey Anti-Rabbit IgG H&L (Alexa FluorⓇ 488) was obtained from Abcam, UK. Donkey Anti-Rabbit IgG H&L (Alexa FluorⓇ 647) was purchased from Abcam, UK.

### 2.3 Dezocine relieves acute morphine withdrawal symptoms

The morphine-dependent rats model was established using a 5-day incremental dose regimen. Morphine was administered subcutaneously three times daily (8:00, 14:00, 20:00) for five consecutive days: 5 mg/kg on Day 1, 10 mg/kg on Day 2, 20 mg/kg on Day 3, 30 mg/kg on Day 4, and 50 mg/kg on Day 5. After the establishment of the model, rats were divided into four groups (*n* = 6): Naive (not administered morphine, no intervention, and subjected only to behavioral observation), DMSO [intraperitoneal injection of equal volume 1% (v/v) DMSO], Bup (buprenorphine hydrochloride 0.3 mg/kg), and Dez (dezocine hydrochloride 1.25 mg/kg). During the 7-day withdrawal-treatment period, rats from each group were placed in an open-field test box daily between 9:00–10:00 a.m. and 4:00–5:00 p.m., and activity videos were recorded. The frequency of acute withdrawal symptoms was documented for each 1-h interval in the morning and afternoon, and scores were assigned based on the modified Maldonado criteria (Maldonado et al., 1992; He et al., 2011). The daily score was the sum of the morning and afternoon scores. The Maldonado scale includes seven indicators. Wet dog shakes, writhing, teeth chattering, jumping, rearing, and body grooming were scored as 1 (1–3 occurrences), 2 (4–6 occurrences), or 3 (≥7 occurrences). Ptosis was scored as 1 (1–4 occurrences), 2 (5–8 occurrences), or 3 (≥9 occurrences). On Day 8, naloxone hydrochloride (2 mg/kg) was administered intraperitoneally at 9:00 a.m. to induce withdrawal symptoms. The Maldonado score was recorded for 1 h at both 9:00 a.m. and 4:00 p.m.

### 2.4 Conditioned place preference (CPP)

#### 2.4.1 CPP model construction and CPP score determination

The CPP model was employed to assess reinstatement behavior (Tzschentke, 2007). Using an XR-XT401 CPP black-and-white training box (Shanghai Xinsoft Information Technology Co., Ltd.). The training box consists of two compartments: a white box and a black box, separated by a partition that allows control over the connectivity between the two compartments. Each compartment measures 18 × 18 × 20 cm^3^. The system is equipped with a camera that provides horizontal resolution of 1,200 lines and a video resolution of 640 × 480 for observation and data collection. Rats underwent an exploratory phase (Day 1–2) to adapt to the environment. During this phase, they were allowed 30 min of activity per session at 9:00 a.m. on Day 1 and 3:00 p.m. on Day 2. The black-and-white compartments were connected, allowing rats to move freely between the two boxes. In the pre-test phase (Day 3–4), rats were tested for natural preferences. During this phase, the compartments remained connected, and each session involved recording 900 s of free movement video. Rats that spent more than 630 s (≥70%of the total test duration) in either compartment were considered to exhibit a strong natural preference and were excluded from the study. In the conditioning phase (Day 5–7), except for the Naive group, all rats received morphine (10 mg/kg) to induce the model, and their activity was recorded. Extinction Phase (Day 11–17) involved daily CPP tests, and if the CPP score was at baseline for three consecutive days, the extinction was confirmed. In the intervention phase (Day 15–17), intervention drugs were administered, and on Day 18, a small dose of morphine (2 mg/kg) was used to assess CPP reinstatement. In this experiment, the Naive group served as the blank control and did not receive any drug interventions.

#### 2.4.2 Effects of κ receptor agonist U50488 and μ receptor antagonist on dezocine blocking CPP reinstatement

To explore the mechanism by which dezocine blocks CPP reinstatement, dezocine was first administered to all rats, followed by subcutaneous injections of different drug combinations in each group: varying concentrations of U50488 (0.33, 1, 3, 5 mg/kg) or a sufficient dose of naloxone (2 mg/kg). These injections were administered immediately after dezocine and continued for three consecutive days during the intervention phase. Following the procedures outlined above, CPP reinstatement was induced in the reinstatement phase by administering 2 mg/kg morphine, and their impact on CPP reinstatement was observed.

### 2.5 Western blot for p-DARPP32 and DDC expression

On Day 18, following behavioral assessment, rats were anesthetized with 2% pentobarbital (0.75 mg/kg) and decapitated. The brains were rapidly removed and exposed on ice, then immediately frozen in liquid nitrogen for 20–25 s. The brains were sectioned coronally into 1 mm thick slices and stored at –80°C until further analysis. A suitable amount of brain tissue was weighed and homogenized in 10 μl/mg RIPA lysis buffer (Bio-Rad, USA), pre-supplemented with 10 μl/ml Halt Protease and Phosphatase Inhibitor Cocktail (100X) (Thermo Scientific™). The tissue was thoroughly homogenized using an ultrasonic homogenizer in an ice bath. The homogenate was then transferred to Eppendorf tubes and incubated on a rotating shaker at 4°C for 2 h to ensure complete lysis. Afterward, the samples were centrifuged at 15,000 rpm for 20 min at 4°C using a high-speed refrigerated centrifuge. The supernatant was collected and stored at –80°C for subsequent analysis. Total protein concentrations in the supernatants were determined using the BCA assay. A total of 4 μl of protein extract was mixed with 1 μl of 5 × protein sample loading buffer (Beyotime Biotechnology, Shanghai). The samples were heated at 98°C for 10 min to denature the proteins. Equal amounts (30 μg) of protein were loaded into each well of an 8% SDS–PAGE gel for separation. Electrophoresis was performed at 90V until the bromophenol blue marker reached ~1 cm from the bottom of the separating gel. The proteins were then transferred to a nitrocellulose membrane at 120 mA for 120 min using a wet transfer system. After the transfer, the membrane was washed three times with TBST for 5 min each time. The membrane was then blocked for 2 h at room temperature in 1% BSA blocking solution (Sigma-Aldrich, USA). After blocking, the membrane was incubated overnight at 4°C with the following primary antibodies: Phospho-DARPP32 (Thr34) polyclonal antibody (PA5-105038, Thermo Fisher Scientific, USA, 1:1,000), Anti-DOPA Decarboxylase antibody (ab211535, Abcam, UK, 1:1,000), or GAPDH Rabbit monoclonal antibody (2118, Cell Signaling Technology, USA, 1:1,000). The membrane was then incubated for 2 h at room temperature with Goat Anti-Rabbit IgG H&L (HRP) (ab6721, Abcam, UK, 1:10,000). After washing, ECL substrate solution was applied to the membrane, and images were captured using a GE ImageQuant LAS 400 digital imaging system. The relative intensity of the bands was analyzed using ImageJ software (version 1.35). The expression levels of target proteins were normalized to the GAPDH intensity. The normalized target protein expression levels were then expressed as a ratio to the average expression level of the naive group.

### 2.6 Immunofluorescence for p-DARPP32 and DDC expression

Post-behavioral testing, rats were anesthetized and perfused with 4°C 1X PBS followed by 4% paraformaldehyde. Brains were then extracted and post-fixed in 30 ml of 4% paraformaldehyde at 4°C overnight. Following fixation, the brains were sequentially dehydrated in 30 ml solutions of 10%, 20%, and 30% sucrose for 24 h each. After the brains were blotted dry with filter paper, they were embedded in OCT compound and frozen at –20°C using a cryostat, then sectioned into 20 μm thick slices. The brain sections were washed three times with 0.3% PBST, followed by permeabilization with 1% PBST for 30 min. Afterward, the sections were washed three times with 0.3% PBST and blocked for 30 min using QuickBlock™ Blocking Buffer for Immunol Staining (P0260, Beyotime, Shanghai). The following primary antibodies were applied for overnight incubation at 4°C: Phospho-DARPP32 (Thr34) polyclonal antibody (PA5-105038, Thermo Fisher Scientific, USA, 1:500) and Anti-DOPA Decarboxylase antibody (ab211535, Abcam, UK, 1:1,000). After washing, sections were incubated with secondary antibodies at room temperature in the dark for 1 h while gently shaking. For detection of Phospho-DARPP32, Donkey Anti-Rabbit IgG H&L (Alexa Fluor 488) (ab150073, Abcam, UK, 1:1,000) was used, and for detection of DOPA Decarboxylase, Donkey Anti-Rabbit IgG H&L (Alexa Fluor 647) (ab150075, Abcam, UK, 1:1,000) was applied. The sections were then washed with 1X PBS and mounted with Antifade Mounting Medium with DAPI (P0131, Beyotime, Shanghai). Sections were visualized under a fluorescence microscope, and during image acquisition, exposure time, brightness, and contrast were adjusted to remain consistent. Quantification of target protein-positive cells was performed using ImageJ software.

### 2.7 Statistics and analysis

Continuous data are presented as the mean ± standard error of the mean (SEM). Statistical analyses were performed using PRISM 10 (Version 10.1.0, released October 18, 2023). Normality of the data in each group was assessed using the Shapiro-Wilk test, and homogeneity of variance was evaluated with the Bartlett test. For repeated measures data, differences were analyzed using a two-way repeated measures ANOVA, with sphericity assessed. If sphericity was not met, the Geisser-Greenhouse correction was applied. *Post hoc* pairwise comparisons were conducted using the Tukey method. For one-way analyses of non-repeated measures data, one-way ANOVA was used, with *post hoc* multiple comparisons conducted using the Tukey method. If the data met normality assumptions but exhibited unequal variances, Welch's ANOVA was used to analyze group differences, followed by Dunnett's T3 multiple comparisons test. Grayscale analysis of Western blot results and positive cell counts from immunofluorescence images were performed using ImageJ (version 1.53t), followed by one-way ANOVA for intergroup comparisons. Data visualization, including figure generation, was completed in PRISM 10. A *p*-value of < 0.05 was considered statistically significant.

## 3 Results

### 3.1 Dezocine relieves withdrawal symptoms of morphine addiction

**Figure 1:**
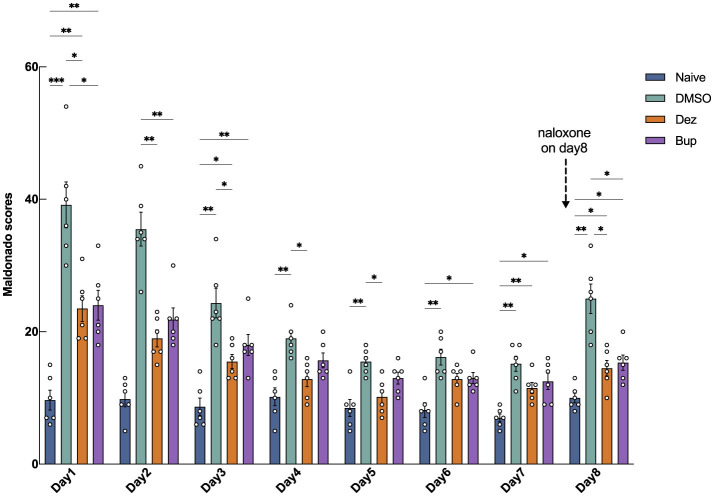
Maldonado withdrawal scores in morphine-addicted rats from day 1 to day 8 following drug withdrawal. During the withdrawal period, the Dez group received dezocine hydrochloride (1.25 mg/kg), the Bup group received buprenorphine hydrochloride (0.3 mg/kg), and the DMSO group received an equal volume of DMSO [1% (v/v)] via intraperitoneal injection, while the naive group underwent no intervention (*n* = 6 per group). On day 8, 2 mg/kg of naloxone was administered to induce withdrawal symptoms. Data were shown as the mean ± standard error of the mean (SEM). *p*-values are indicated as follows: ^*^p < 0.05, ^**^p < 0.01, ^***^p < 0.001. Statistical analysis showed significant effects of group (*F* = 62, dF = 3, 20, *p* < 0.001), time (*F* = 44, dF = 4.1, 82, *p* < 0.001), and their interaction (*F* = 6.9, dF = 21, 140, *p* < 0.001) on the outcome, indicating substantial effects of drug intervention, time, and their combined influence.

The results of morphine-induced withdrawal in rats are presented in Figure 1. The group effect was significant (*F* = 62, dF = 3, 20, *p* < 0.001), indicating that drug intervention within different groups significantly impacted the outcome. The time effect was also significant (*F* = 44, dF = 4.1, 82, *p* < 0.001), suggesting a strong influence of study days on the outcome. The interaction between group and time was significant (*F* = 6.9, dF = 21, 140, *p* < 0.001), highlighting a significant combined effect of group and day on the outcome. On the first day of drug withdrawal, the Maldonado scores in the DMSO group (30, 95% CI: 17 to 42, ^***^p < 0.001), Dez group (14, 95% CI: 6.3 to 21, ^**^p < 0.01), and Bup group (14, 95% CI: 5.8 to 23, ^**^p < 0.01) were significantly higher compared to the Naive group, indicating successful induction of withdrawal symptoms. The Maldonado scores in the Dez group were significantly lower than those in the DMSO group on day 1 (–16, 95% CI: –28 to –2.9, ^*^p < 0.05 ), day 2 (–17, 95% CI: –26 to –7.1, ^**^p < 0.01), day 3 (–8.8, 95% CI: –17 to –0.6, ^*^p < 0.05), day 4 (–6.2, 95% CI: –11 to –1.2, ^*^p < 0.05), and day 5 (–5.3, 95% CI: –9.5 to –1.2, ^*^p < 0.05). Compared to the Naive group, the Dez group showed no significant differences on days 4 and 5. By day 6, no significant differences were observed between the Dez group and either the DMSO or Naive groups. Similarly, the Maldonado scores in the Bup group were significantly lower than those in the DMSO group on day 1 (–15, 95% CI: –28 to –2.1, ^*^p < 0.05), day 2 (–14, 95% CI: –23 to –3.9, ^**^p < 0.01). By day 3, no significant differences were observed between the Bup group and the DMSO group. However, no significant difference was observed between the Dez group and the Bup group during the experiment.

On day 8, after naloxone-induced withdrawal, the Dez and the Bup groups all exhibited a slight increase (Dez vs. Naive, 4.5, 95% CI: 0.11 to 8.9, ^*^p < 0.05; Bup vs. Naive, 5.3, 95% CI: 1.0 to 9.6, ^*^p < 0.05) and there was no significant difference between the Dez and Bup groups. However, both the Dez and Bup groups demonstrated significantly lower scores compared to the DMSO group (Dez vs. DMSO, –11, 95% CI: –19 to –2.4, ^*^p < 0.05; Bup vs. DMSO, –2.7, 95% CI: –18 to –1.5, ^*^p < 0.05). This suggested that, at the given concentration, dezocine exhibited a similar ability to buprenorphine in alleviating morphine withdrawal symptoms in rats.

### 3.2 Effects of κ receptor activation and μ receptor antagonism on the reactivation of CPP blocked by dezocine

Dezocine exhibits dual pharmacological actions: it antagonizes the κ receptor and agonizes the μ receptor. However, it remains unclear which receptor activity primarily mediates the blockade of CPP reinstatement in rats. To address this, we explored the specific pharmacological mechanism underlying dezocine's effects using two distinct approaches: administering the κ receptor agonist U50488 to counteract dezocine's κ receptor antagonism and administering naloxone to antagonize dezocine's μ receptor agonist effect.

**Figure 2:**
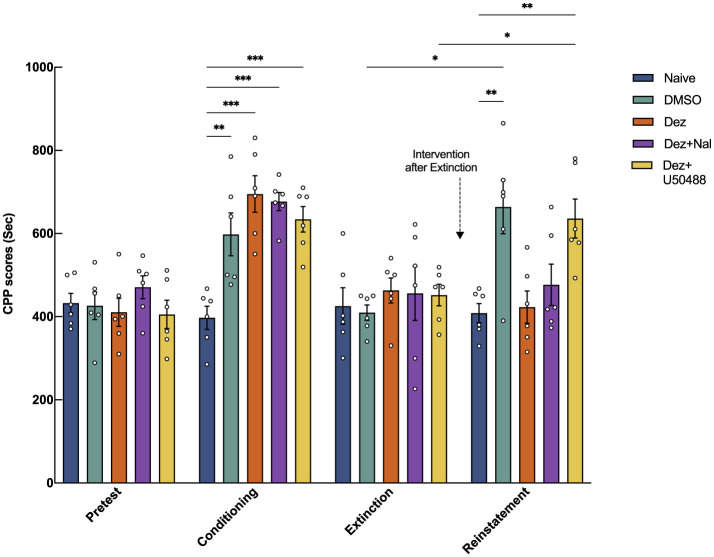
Effects of intraperitoneal injection of the μ-opioid receptor antagonist naloxone and the κ-opioid receptor agonist U50488 on the blockade of CPP reinstatement by dezocine. CPP scores were measured during the pre-test phase (Days 3–4), conditioning phase (Days 5–7), extinction phase (Days 11–17), and reinstatement phase (Day 18). Intervention drugs were administered during the intervention phase (Days 15–17). On Day 18, a small dose of morphine (2 mg/kg) was administered to assess CPP reinstatement (*n* = 6 per group). Data were shown as the mean ± standard error of the mean (SEM). *p*-values are indicated as follows: ^*^p < 0.05, ^**^p < 0.01, ^***^p < 0.001. Statistical analysis showed significant effects of group (*F* = 3.4, dF = 4, 25, *p* < 0.05), phase (*F* = 27, dF = 2.6, 65, *p* < 0.001), and their interaction (*F* = 6.0, dF = 12, 75, *p* < 0.001) on the outcome, indicating substantial effects of drug intervention, phase, and their combined influence.

We further compared the effects of dezocine combined with the κ receptor agonist U50488 and dezocine combined with the μ receptor antagonist naloxone on morphine-induced reinstatement of CPP (*n* = 6; Figure 2). After confirming the extinction of CPP during the intervention phase, rats received their respective treatments intraperitoneally for three consecutive days. Statistical analysis showed significant effects of group (*F* = 3.4, dF = 4, 25, *p* < 0.05), phase (*F* = 27, dF = 2.6, 65, *p* < 0.001), and their interaction (*F* = 6.0, dF = 12, 75, *p* < 0.001) on the outcome, indicating substantial effects of drug intervention, phase, and their combined influence. Results showed that following the administration of a small dose of morphine during the reinstatement phase, the CPP scores in the DMSO group and the Dez + U50488 group were significantly higher compared to the Naive group (DMSO vs. Naive: 225, 95% CI: 90 to 421, *p* < 0.01; Dez + U50488 vs. Naive: 228, 95% CI: 105 to 350, *p* < 0.001), indicating reinstatement of CPP behavior in these groups. However, no significant difference was observed between the DMSO group and the Dez + U50488 group. In contrast, the Dez group showed no significant difference in CPP scores compared to the Naive group, a finding that was also observed in the Dez + Naloxone group. Notably, the administration of naloxone during the intervention phase did not attenuate the effect of dezocine on CPP reinstatement, as no significant difference was observed between the Dez + Naloxone group and the Dez group (54, 95% CI: —88 to 195, *p* < 0.01). Finally, the CPP scores in the Dez + U50488 group were significantly higher than those in the Dez group during the reinstatement phase (213, 95% CI: 77 to 349, *p* < 0.01), suggesting that U50488 reversed the effect of dezocine on CPP reinstatement (Figure 2, Supplementary Figure S1). Our results indicate that the effect of dezocine in alleviating the reinstatement of CPP was attenuated by U50488. However, treatment with a sufficient dose of naloxone did not weaken this effect of dezocine. Therefore, we hypothesize that dezocine's ability to mitigate CPP reinstatement may be attributed to its inherent KOR antagonistic properties.

### 3.3 Western blot analysis of p-DARPP32 and DDC expression in the NAc

**Figure 3:**
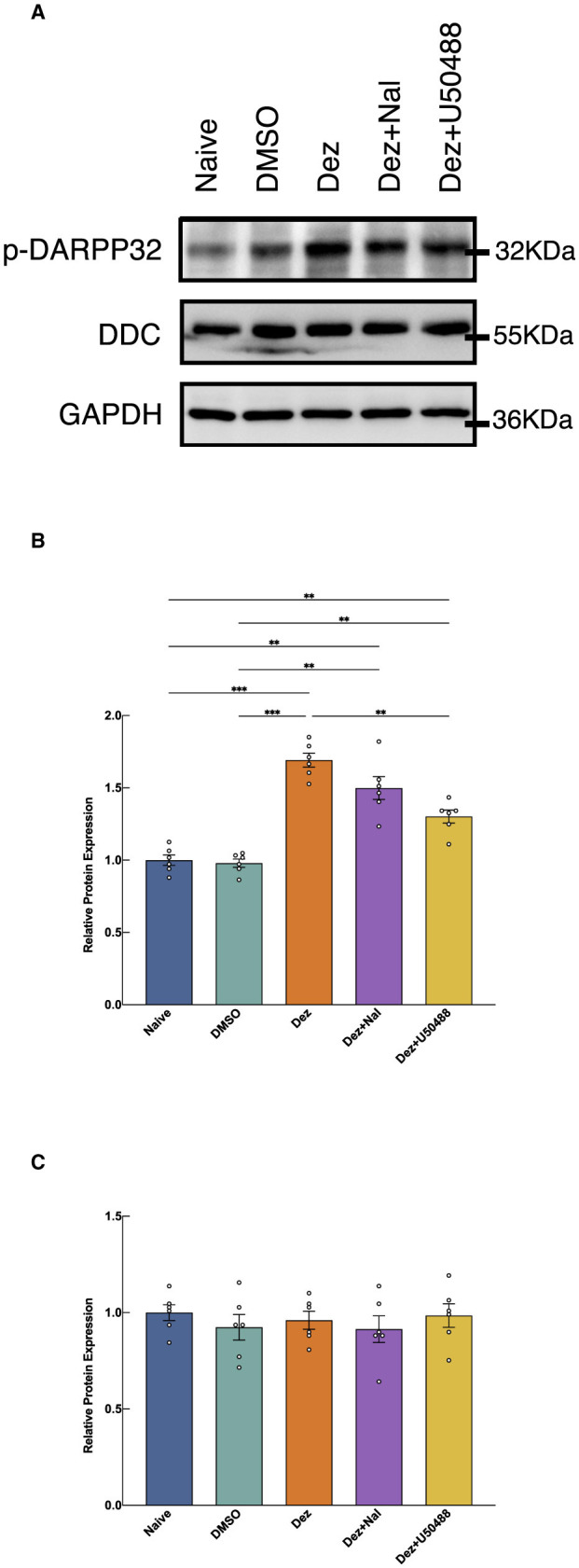
Expression levels of p-DARPP32 and DDC in the NAc, detected by Western blotting. **(A)** The expression levels of p-DARPP32 and DDC were shown (*n* = 6 per group). **(B)** The results show the expression of p-DARPP32 in the NAc of rats from each group, normalized to the GAPDH intensity. The normalized target protein expression levels were then expressed as a ratio to the average expression level of the naive group. Welch's ANOVA revealed a significant difference among group means (*W* = 45, dF = 4.0, 12, *p* < 0.001), indicating that the group effects were statistically significant. **(C)** The expression of DDC in the NAc of rats from each group, normalized to the GAPDH intensity. The normalized target protein expression levels were then expressed as a ratio to the average expression level of the naive group. The ANOVA analysis showed no significant difference between treatment groups (*F* = 0.4152, dF = 4, 25, *p* = 0.796). Data were shown as the mean ± standard error of the mean (SEM). *p*-values are indicated as follows: ^**^p < 0.01, ^***^p < 0.001. **(A)** Western blot results of p-DARPP32 and DDC expression in the nucleus accumbens (NAc). **(B)** Western Blot Analysis of p-DARPP32 in NAc. **(C)** Western Blot Analysis of DDC in NAc.

Western blot analysis was performed to assess the expression of p-DARPP32 and DDC in the nucleus accumbens (NAc) of rat brains across different experimental groups (*n* = 6; Figure 3). Welch's ANOVA revealed a significant difference among group means (*W* = 45, dF = 4.0, 12, *p* < 0.001). *Post hoc* multiple comparisons revealed that the expression of p-DARPP32 was significantly elevated in the Dez group compared to the DMSO group (0.71, 95% CI: 0.51 to 0.92, *p* < 0.001), a trend also observed in the Dez + Nal (0.52, 95% CI: 0.18 to 0.86, *p* < 0.01) and Dez + U50488 groups (0.32, 95% CI: 0.13 to 0.52, *p* < 0.001). No significant difference was found between the Dez + Nal group and the Dez group. However, the Dez + U50488 group showed a significant decrease in p-DARPP32 expression compared to the Dez group (0.39, 95% CI: 0.16 to 0.62, *p* < 0.001; Figure 3C) These results indicate a marked enhancement of dopamine response in the NAc across all three intervention groups. Furthermore, U50488 administration following dezocine treatment attenuated dopamine response in the NAc, whereas naloxone intervention had no significant impact on dopamine response intensity after dezocine treatment.

As for DDC expression, no statistically significant differences were observed in the relative expression levels among the groups in the NAc (*F* = 0.4152, dF = 4, 25, *p* = 0.796; Figure 3C). These findings suggest that dezocine does not significantly alter DDC expression levels in the NAc, indicating that its modulatory effects on dopamine-related signaling may not involve changes in DDC-mediated dopamine synthesis.

### 3.4 Immunofluorescence assay of p-DARPP32 and DDC expression in reward-related nuclei

**Figure 4:**
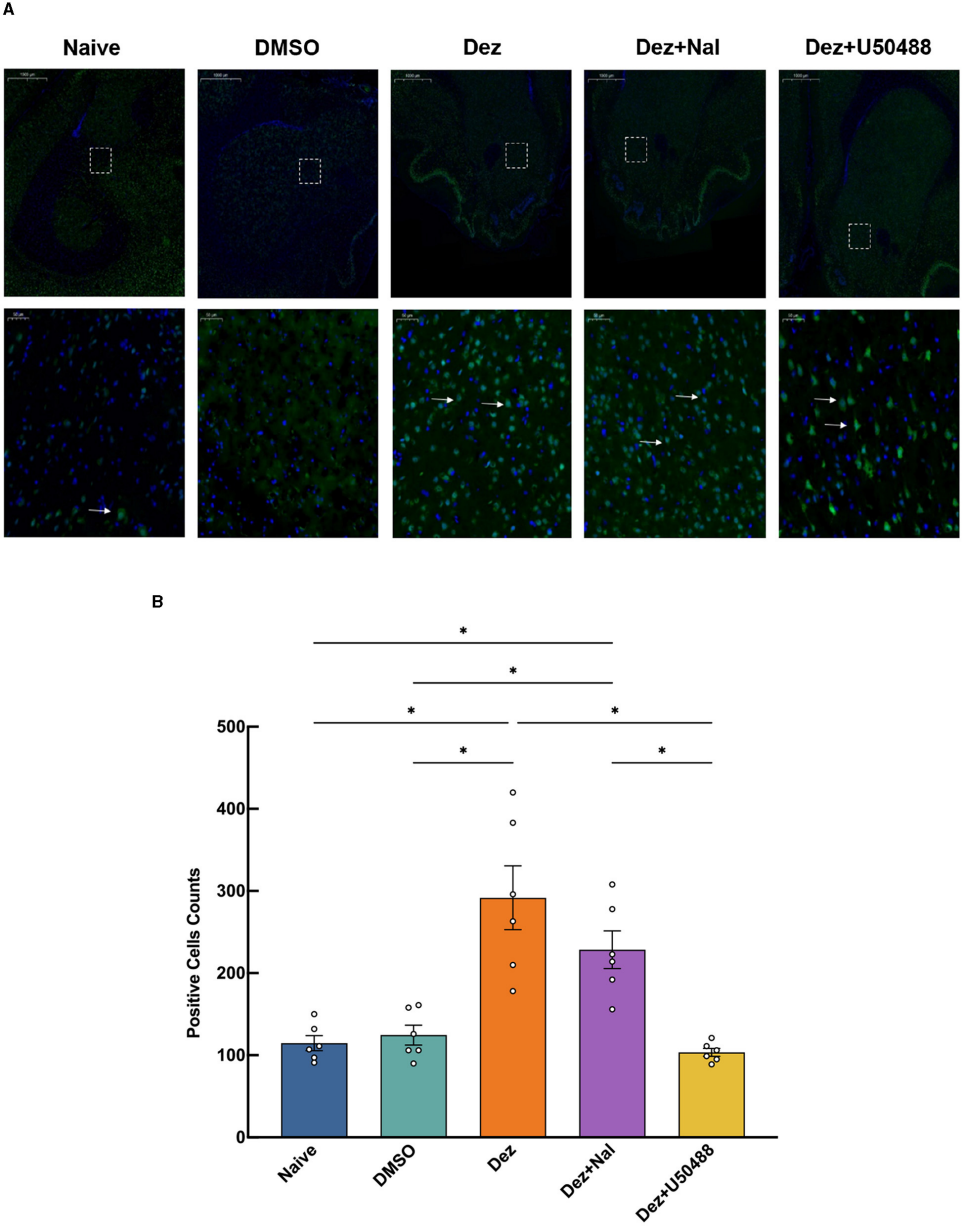
Expression of p-DARPP32 positive cells in the NAc for each group(*n* = 6 per group). **(A)** The upper row shows low magnification images, while the lower row provides a detailed view of the positive cell expression within the dashed white box. White arrows indicate p-DARPP32 positive cells.**(B)** Counting results of p-DARPP32 positive cells in the NAc. Data were shown as the mean ± standard error of the mean (SEM). *p*-values are indicated as follows: ^*^p < 0.05. **(A)** Expression of p-DARPP32 positive cells in the NAc, detected by immunofluorescence. **(B)** Counting of p-DARPP32 positive cells in the NAc.

**Figure 5:**
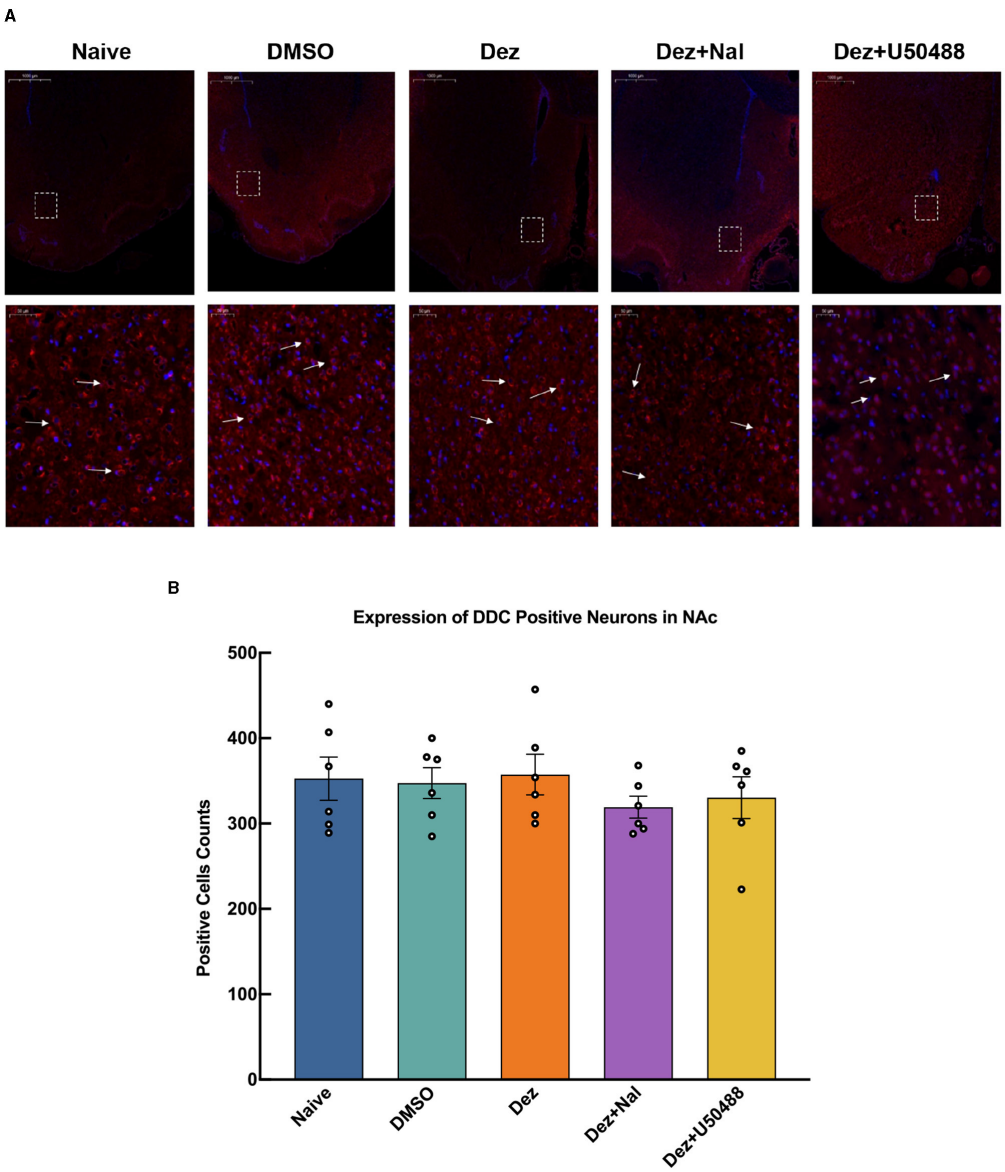
Expression of DDC positive cells in the NAc for each group (*n* = 6 per group). **(A)** The upper row shows low magnification images, while the lower row provides a detailed view of the positive cell expression within the dashed white box. White arrows indicate DDC positive cells. **(B)** DDC expression in the NAc was assessed through immunofluorescence. Statistical analysis showed no significant differences in the number of DDC-positive cells among the groups (*F* = 0.5, dF = 4, 25, *p* = 0.693). Data were shown as the mean ± standard error of the mean (SEM). **(A)** Expression of DDC positive cells in the NAc, detected by immunofluorescence. **(B)** Counting of DDC positive cells in the NAc.

An immunofluorescence assay was conducted to evaluate changes in the number of p-DARPP32-positive cells within reward-related brain regions, including NAc, ventral tegmental area (VTA), hippocampus (HP), and prefrontal cortex (PFC), in the rats brain (*n* = 6). In the NAc, Welch's ANOVA revealed a significant difference among group means (*W* = 11, dF = 4.0, 11, *p* < 0.001). p-DARPP32 expression in the Dez group was significantly higher compared to the Naive group (177, 95% CI: 17 to 337, *p* < 0.001). Likewise, the Dez+Nal group exhibited significantly higher p-DARPP32 expression than the Naive group (114, 95% CI: 20 to 207, *p* < 0.05). Following U50488 intervention, p-DARPP32 expression in the NAc showed no significant difference from the Naive group and was significantly lower than in the Dez group (–188, 95% CI: –357 to –19, *p* < 0.001; Figure 4). This pattern was similarly observed in the VTA, HP, and PFC (Supplementary Figures S2–S4). Additionally, DDC expression in the NAc was assessed through immunofluorescence. Statistical analysis indicated no significant differences in the number of DDC-positive cells among the groups (*F* = 0.5, dF = 4, 25, *p* = 0.693; Figure 5). These results were consistent with the findings from Western blot analysis, suggesting that dezocine selectively modulates dopamine signaling without affecting DDC-mediated dopamine synthesis.

## 4 Discussion

In this study, we demonstrated that dezocine significantly reduces morphine-induced CPP reinstatement in rats, highlighting its potential as a therapeutic agent for opioid addiction. Our findings indicate that dezocine not only alleviates withdrawal symptoms but also reverses morphine-induced CPP reinstatement. Notably, this effect was reversed by the KOR agonist U50488 but not by the μ-opioid receptor (MOR) antagonist naloxone, suggesting that dezocine's therapeutic effects are primarily mediated through KOR antagonism rather than MOR pathways. Furthermore, dezocine's impact appears closely associated with its influence on the brain's reward circuitry, as evidenced by the significant upregulation of p-DARPP32 in key reward-related regions, including the VTA, NAc, PFC, and HP (John et al., 2020).

A key finding of our study is that dezocine's attenuation of morphine-induced CPP reinstatement is likely mediated through KOR antagonism. Previous research has established the critical role of KORs in opioid addiction and reinstatement (Spanagel et al., 1994). KOR activation suppresses dopamine release in reward-related brain regions such as the VTA and NAc by engaging mitogen-activated protein kinase, thereby decreasing reward system excitability and inducing aversive behaviors in rodents (Benjamin et al., 2009; Jonathan et al., 2015). Similarly, the study by Abraham et al. produced results consistent with ours, demonstrating that KOR agonists induced compulsive behavior, while systemic KOR antagonists could prevent stress-induced disruptions in performance on the differential reinforcement of low response rate task, thereby inhibiting the “burst” of incorrect responses caused by KOR activation (Antony et al., 2018). Recent study by Brooks et al. have demonstrated that KOR activation inhibits synaptic function in the hippocampus, reducing synaptic electrophysiological activity and neurotransmitter release (Julie and Patricio, 2017). Conversely, KOR activation impairs dopamine release in the NAc and caudate putamen, leading to negative emotional states such as loss of pleasure, anxiety, and restlessness (Mykel et al., 2020). Mice lacking the dynorphin gene do not exhibit increased CPP in response to stress or cocaine conditioning, whereas wild-type mice show significant CPP enhancement (Van and Charles, 2008), underscoring the role of dynorphin/KOR interactions in addiction and stress responses. This adaptive mechanism protects against potential brain damage from excessive dopamine release mediated by MOR activation during opioid addiction cycles. By antagonizing KORs, dezocine may enhance dopaminergic signaling. Previous studies have shown that downregulating the dynorphin/KOR system reduces withdrawal symptoms and aversive behaviors, a result linked to changes in mesolimbic system function and dopamine levels (Mykel et al., 2020). This downregulation complements the regulation of dopamine neurotransmission in the midbrain reward system and explains the high expression of KORs on dopaminergic neuron axons observed in neuroanatomical studies. Additionally, the downregulation of dynorphin/KOR activity, combined with MOR excitation, inhibits GABAergic interneurons in the NAc and VTA, reducing neurotransmitter release and producing a disinhibition effect that enhances reward system excitability and dopamine overactivity (Van and Charles, 2008). Our data showed that the KOR agonist U50488 reversed dezocine's therapeutic effects, consistent with the hypothesis that dezocine's actions are mediated by KOR inhibition. This finding aligns with previous studies indicating that KOR antagonism may reduce opioid-seeking behavior by enhancing reward system function. The inability of naloxone to reverse dezocine's effects further supports the notion that dezocine's mechanism is independent of MOR pathways.

Our results suggest that dezocine's efficacy in reversing morphine-induced CPP reinstatement involves the activation of key reward-related brain regions. Notably, we observed a significant increase in the expression of p-DARPP32 in the VTA, NAc, PFC, and HP in the dezocine-treated group. DARPP32 is a crucial mediator of dopaminergic signaling, with its phosphorylation indicating increased dopamine receptor activity (Fienberg et al., 1998). DARPP32 is prominently expressed in brain regions rich in dopaminergic neuron projections, including the VTA, NAc, PFC, and HP (Arlene et al., 2023), which are essential for reward processing. Alterations in dopamine signaling within these areas influence reward thresholds and addiction-related behavior. In animals where CPP reinstatement was effectively blocked, enhanced dopamine responses were observed throughout the primary dopamine reward circuitry, elevating reward thresholds and preventing CPP reinstatement (Shiwei et al., 2019). As a result, a minimal dose of morphine used to induce CPP in the Dez and Dez+Nal groups was ineffective in reinstating CPP. Additionally, activation of KORs by U50488 led to a significant decrease in p-DARPP32 expression, underscoring the critical role of KOR antagonism by dezocine in enhancing reward system excitability. This observation aligns with the established effects of KOR activation, which include reduced dopamine release and diminished reward sensitivity.

To explore the potential mechanism by which dezocine alleviates CPP reinstatement, we examined DARPP-32 and DDC expression in reward-related brain regions. DARPP-32, a dopamine- and cAMP-regulated phosphoprotein, is a primary target of adenylate cyclase. Dopamine receptor activation promotes the phosphorylation of DARPP-32, making it a key marker of dopaminergic activity (Scheggi et al., 2018). Previous studies have shown that dopamine receptor-mediated cellular responses influence reward effects and subsequently affect behaviors associated with substance abuse and drug-seeking tendencies (Volkow et al., 2019b). Given the close interaction between KOR and dopamine, we initially hypothesized that dezocine might mitigate CPP reinstatement by modulating dopaminergic activity within the mesencephalon through its effects on DARPP-32 expression. Moreover, existing research has suggested a potential link between drug abuse and DARPP-32. For example, drug abuse may alter phosphorylation at the Thr34 site of DARPP-32, activating downstream ERK pathways and influencing behaviors such as psychostimulant-induced locomotor sensitization (Valjent et al., 2005). Another study on morphine-induced sensitization demonstrated that morphine challenges alter phosphorylation states at both the Thr34 and Thr75 sites of DARPP-32, which may represent one mechanism underlying morphine-induced sensitization (Scheggi et al., 2009). Dopamine elevation induced by drug abuse may also lead to DARPP-32 enrichment in target cells, particularly in the nucleus accumbens (NAc) (Di Chiara, 1999). Additionally, DARPP-32 knockout mice exhibit reduced cocaine-induced CPP, highlighting the potential role of DARPP-32 phosphorylation in drug abuse-related behaviors (Zachariou et al., 2006). Our findings present an intriguing deviation from prior studies. We observed that dezocine increased phosphorylation of DARPP-32 at the Thr34 site in the reward circuitry; however, rats exhibited suppression, rather than reinstatement, of CPP behavior. A study has suggested that dopamine release in the NAc may contribute to the reinstatement of opioid- and psychostimulant-seeking behaviors induced by drugs (Self, 1998). Our finding appears contradictory to previous study, which may be attributed to differences in experimental design and the distinct pharmacological mechanism of dezocine. Dezocine may enhance dopaminergic responses within the brain's reward system, raising the reward threshold. Consequently, conditional or cue-related stimuli during the CPP reinstatement test may fail to elicit further activation of the reward system, resulting in an observable suppression of CPP reinstatement. Interestingly, this phenomenon aligns with the concept of reward pathway saturation proposed in earlier research (Self, 1998). In our study, small doses of morphine failed to induce CPP reinstatement in the Dezocine or Dezocine + Naloxone groups, suggesting that dezocine's modulatory effect may involve altering dopaminergic response thresholds to prevent further reward system activation.

To explore whether dezocine enhances dopamine synthesis in the brain, given the observed increase in dopaminergic activity, we assessed the expression of DDC, the rate-limiting enzyme in dopamine biosynthesis within the nervous system. Alterations in DDC expression reflect changes in dopamine synthesis (Li et al., 2020). Our initial hypothesis proposed that increased dopamine synthesis might underlie the enhanced dopaminergic response observed in our study. We measured DDC expression in the NAc, a central integrative nucleus of the reward system, using Western blot and immunofluorescence techniques. The results demonstrated no statistically significant differences in DDC levels among the Dez, Dez + Nal, Dez + U50488, DMSO, and Naïve groups. This indicates that dopamine synthesis was not elevated in the dopaminergic neurons of the reward system following intervention. Although our study did not investigate dopamine metabolism or degradation, two key findings allow us to propose an alternative mechanism: the presence of enhanced dopamine response in the reward system and the lack of differences in DDC levels in the NAc across groups. We hypothesize that the increased dopaminergic response mediated by KOR antagonism may result from enhanced dopamine release from intracellular stores in dopaminergic neurons, rather than increased dopamine synthesis. KORs, part of the G-protein-coupled receptor superfamily, regulate downstream signaling pathways and membrane Ca^2+^ channel activity via second messenger modulation upon ligand binding (Han et al., 2023). Activation of presynaptic KORs in the central nervous system inhibits Ca^2+^ influx through voltage-dependent channels, reducing neurotransmitter release (Rusin et al., 1997). Studies on dopamine transport inhibitors have shown that the KOR agonist U50488 significantly reduces dopamine release without affecting reuptake (Hoffman et al., 2016). This may explain the absence of increased DARPP-32 Thr34 phosphorylation and the diminished therapeutic effect of dezocine when administered after U50488 treatment. Based on the current evidence, dezocine may enhance dopamine response during CPP reinstatement by antagonizing KORs in the reward system, reducing the closure of membrane Ca^2+^ channels, increasing Ca^2+^ influx at presynaptic terminals, and facilitating dopamine release. However, this hypothesis remains contentious. One study reported that the selective KOR antagonist nor-binaltorphimine failed to block U50488-induced dopamine release reduction in cell experiments (Chudapongse et al., 2003). Further studies are required to elucidate the precise role of KOR activation in modulating dopaminergic responses.

It is necessary to clarify the different opioid-modulating drugs used in this study, as their effects may limit the generalizability of our conclusions and indicate a need for further investigation. In experiments addressing withdrawal symptoms and CPP reinstatement in morphine-exposed rats, we used interventions of 0.3 mg/kg buprenorphine and 1.25 mg/kg dezocine. According to reports, low-dose intravenous administration of buprenorphine at 0.3 mg/kg effectively alleviates opioid withdrawal syndrome in morphine-exposed mice (Lepore et al., 2023). Additionally, experiments examining buprenorphine-induced CPP revealed an inverted U-shaped dose-response curve, indicating that at doses of 0.1 and 1.0 mg/kg, buprenorphine exhibited a rewarding effect, inducing CPP behavior (Tzschentke, 2004). Based on these findings and our previous experimental results, we selected 0.3 mg/kg as the experimental dose of buprenorphine. Dezocine, however, was used at a dose four times that of buprenorphine due to its lower receptor affinity, and its selection was also informed by our previous research on dezocine's effects in alleviating morphine withdrawal. As is well known, Buprenorphine has a higher affinity for the MOR and differs from dezocine in terms of bioavailability, half-life, and brain permeability. Therefore, it would be unsound to directly compare the efficacy of one drug over the other based solely on their observed effects. Consequently, we cautiously concluded that under our experimental conditions, dezocine exhibited effects similar to those of buprenorphine. To explore whether dezocine's effects are primarily mediated through MOR agonism or KOR antagonism, we used naloxone, a clinically common opioid receptor antagonist for reversing opioid overdose, at a higher dose of 2 mg/kg to fully block MOR activation (Kang et al., 2022). Although naloxone primarily targets the MOR, it also possesses some KOR antagonistic properties. When combined with dezocine, naloxone fully antagonized the MOR, and may have interacted synergistically with dezocine in blocking the KOR. While this interaction may have influenced the results, it also supports the conclusion that dezocine's effects are not solely mediated by MOR agonism. However, we acknowledge this limitation to clarify our study for the reader. For future experiments, cyprodime (Chen et al., 1993), a selective MOR antagonist that crosses the blood-brain barrier, may be more suitable for further exploring the underlying mechanisms.

In our study, dezocine, through its antagonism of KORs and subsequent enhancement of reward system activity, may represent a novel approach to treating opioid addiction. The ongoing opioid crisis has exposed the limitations of current treatments, particularly in terms of preventing reinstatement. However, due to the constraints of this study, we have not fully elucidated the precise mechanisms by which dezocine influences the dopamine reward system, nor have we determined if its effects are exclusively mediated through KORs. Further research is required to comprehensively understand the neurobiological mechanisms underlying dezocine's effects. Future studies should also investigate the broader impact of dezocine on other neurotransmitter systems involved in addiction, such as glutamate and GABA, to gain a more thorough understanding of its therapeutic potential. Additionally, clinical trials are essential to validate our preclinical findings and evaluate the safety and efficacy of dezocine in human populations suffering from opioid addiction.

In conclusion, this study reveals that dezocine significantly reverses morphine-induced CPP reinstatement in rats through KOR antagonism and the enhancement of dopaminergic signaling in reward-related brain regions. These findings suggest that dezocine may be a promising novel therapeutic agent for opioid addiction. Further research is needed to investigate its clinical potential and the underlying molecular mechanisms.

## Data Availability

The raw data supporting the conclusions of this article will be made available by the authors, without undue reservation.
