# Supplementary material for: Dezocine modulates the reinstatement of conditioned place preference in morphine-dependent rats via the dopamine reward circuitry
Source: Front Neurosci. 2025 Feb 18;19:1507747. doi: 10.3389/fnins.2025.1507747 (PMC11876162; doi:10.3389/fnins.2025.1507747)
Supplement: Supplementary file 1 [file Data_Sheet_1.pdf]

Supplementary Material

1 SUPPLEMENTARY TABLES AND FIGURES

1.1 Figures

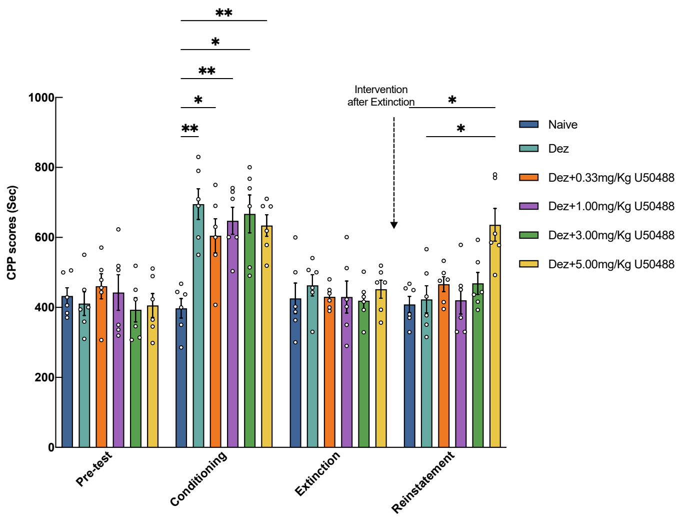

Figure S1. Effects of intraperitoneal injection of U50488 on the blockade of CPP reinstatement. CPP scores during the pre-test phase (Day 3-4), conditioning phase (Day 5-7), extinction phase (Day 11-17), and reinstatement phase(Day 18). Intervention drugs were administered during the intervention phase (Day 15-17). On Day 18, a small dose of morphine (1–2 mg/kg) was administered to assess CPP reinstatement. Statistical analysis showed significant effects of group (F = 2.2, df = 5, 30, $p = 0.078$), phase (F = 47, df = 2.7, 82, $p < 0.001$), and their interaction (F = 5.1, df = 15, 90, $p < 0.001$) on the outcome, indicating substantial effects of drug intervention, phase, and their combined influence. P values are indicated as follows: $*P < 0.05$, $**P < 0.01$, $***P < 0.001$.

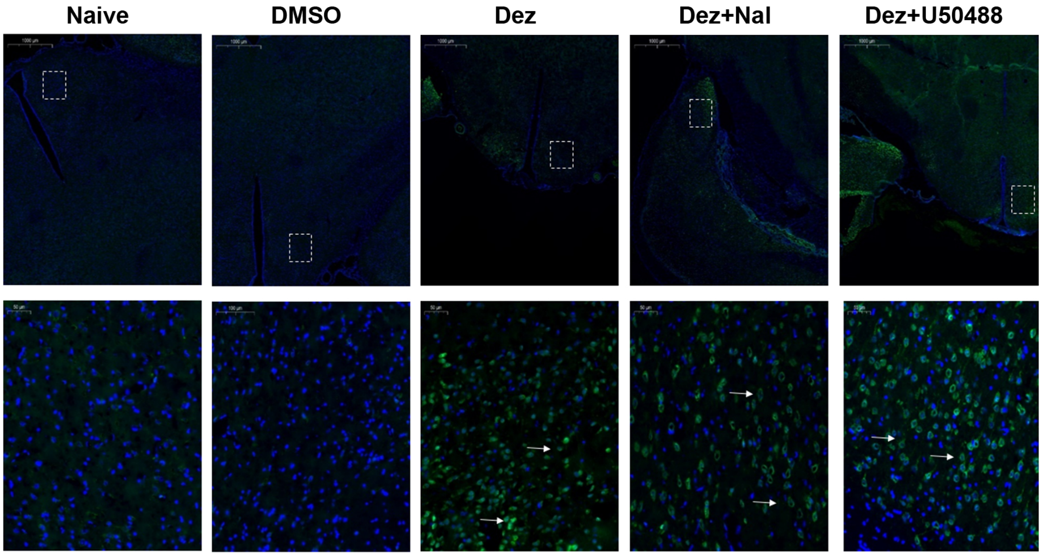

Figure 2a. Expression of p-DARPP32 positive cells in the VTA, detected by immunofluorescence.

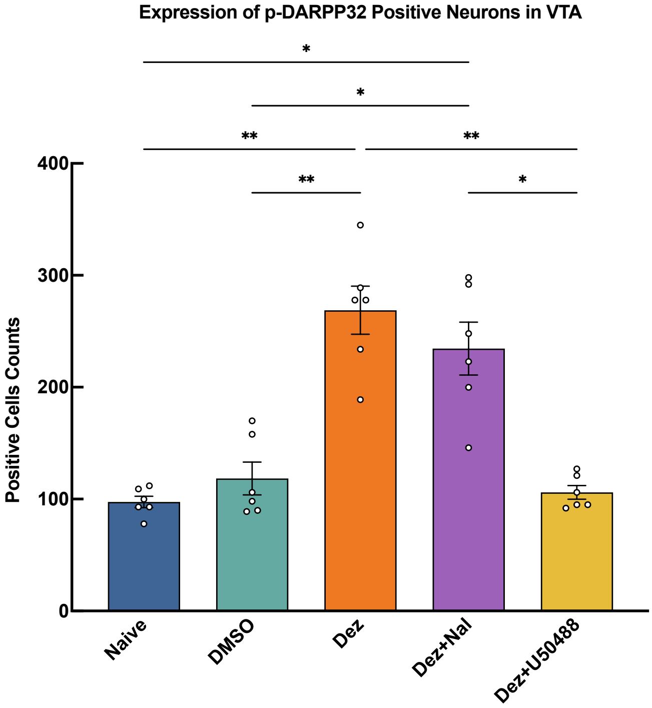

Figure 2b. Counting of p-DARPP32 positive cells in the VTA.

Figure 2. Expression of p-DARPP32 positive cells in the VTA for each group. **(A)**The upper row shows low magnification images, while the lower row provides a detailed view of the positive cell expression within the dashed white box. White arrows indicate p-DARPP32 positive cells.**(B)**Counting results of p-DARPP32 positive cells in the VTA. P values are indicated as follows: $*P < 0.05$, $**P < 0.01$, $***P < 0.001$.

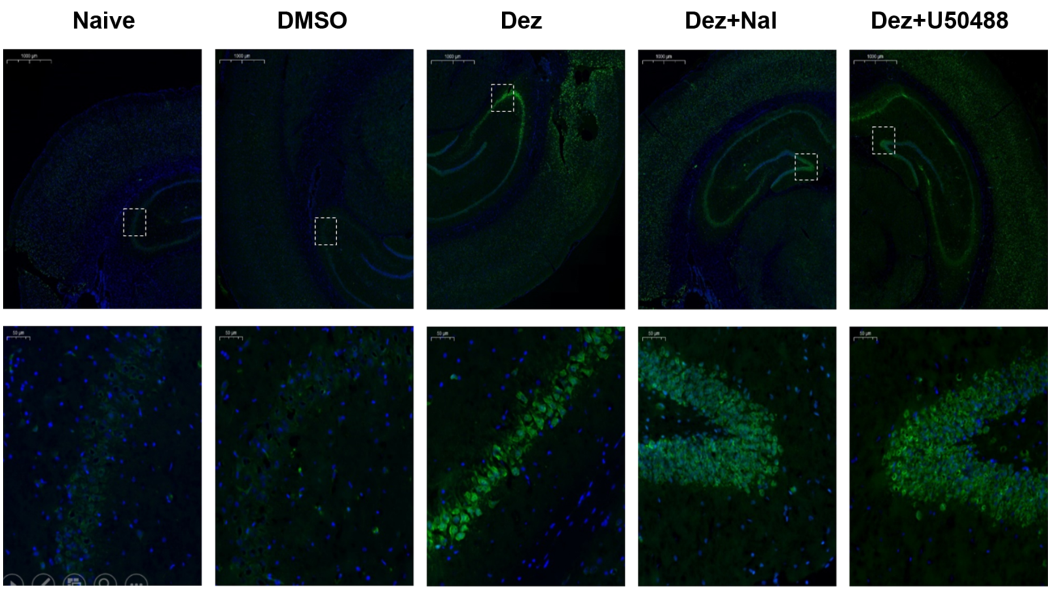

Figure 3a. Expression of p-DARPP32 positive cells in the HP, detected by immunofluorescence.

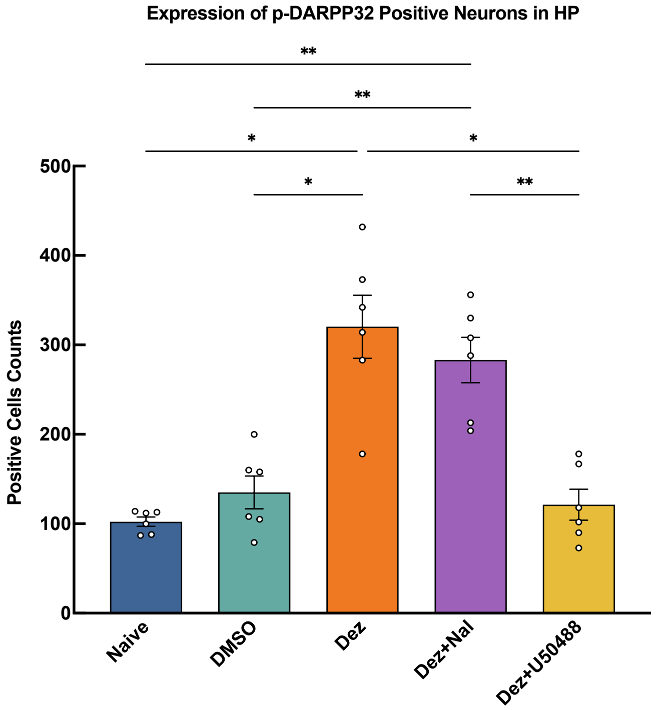

Figure 3b. Counting of p-DARPP32 positive cells in the HP.

Figure 3. Expression of p-DARPP32 positive cells in the HP for each group. **(A)**The upper row shows low magnification images, while the lower row provides a detailed view of the positive cell expression within the dashed white box. White arrows indicate p-DARPP32 positive cells.**(B)**Counting results of p-DARPP32 positive cells in the HP. P values are indicated as follows: $*P < 0.05$, $**P < 0.01$, $***P < 0.001$.

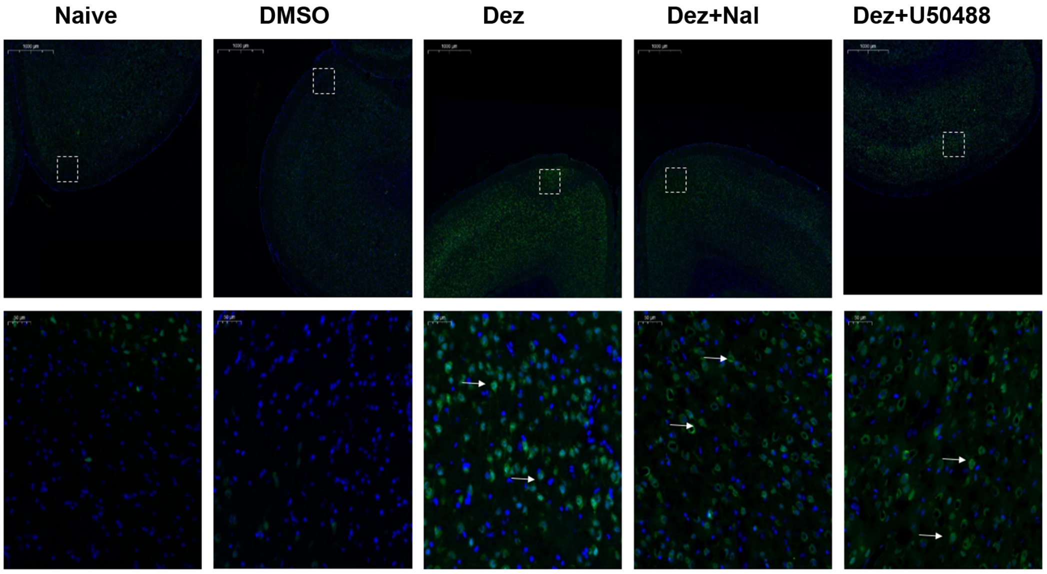

Figure 4a. Expression of p-DARPP32 positive cells in the PFC, detected by immunofluorescence.

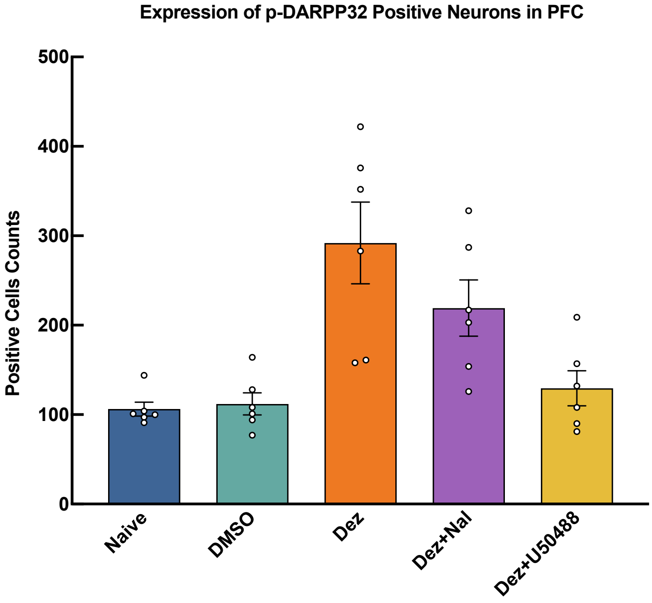

Figure 4b. Counting of p-DARPP32 positive cells in the PFC.

Figure 4. Expression of p-DARPP32 positive cells in the PFC for each group. (**A**)The upper row shows low magnification images, while the lower row provides a detailed view of the positive cell expression within the dashed white box. White arrows indicate p-DARPP32 positive cells.(**B**)Counting results of p-DARPP32 positive cells in the PFC. P values are indicated as follows: $*P < 0.05$, $**P < 0.01$, $***P < 0.001$.

Modified Maldonado Score

Behavior	Score 1 (Mild)	Score 2 (Moderate)	Score 3 (Severe)
Wet dog shakes	1 – 3 occurrences	4 – 6 occurrences	≥ 7 occurrences
Teeth chattering	1 – 3 occurrences	4 – 6 occurrences	≥ 7 occurrences
Jumping	1 – 3 occurrences	4 – 6 occurrences	≥ 7 occurrences
Rearing	1 – 3 occurrences	4 – 6 occurrences	≥ 7 occurrences
Body grooming	1 – 3 occurrences	4 – 6 occurrences	≥ 7 occurrences
Ptosis	1 – 4 occurrences	5 – 8 occurrences	≥ 9 occurrences

Figure S5. Caption

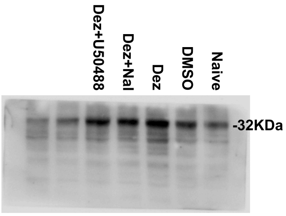

Figure S6. Expression of p-DARPP32 in NAc

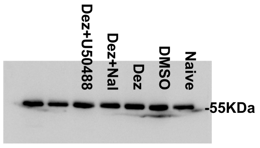

Figure S7. Expression of DDC in NAc

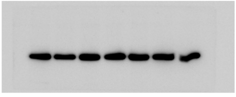

Figure S8. Expression of GAPDH in NAc
